# DNA methylation-based age estimation for adults and minors: considering sex-specific differences and non-linear correlations

**DOI:** 10.1007/s00414-023-02967-6

**Published:** 2023-02-22

**Authors:** Laura Carlsen, Olivia Holländer, Moritz Fabian Danzer, Marielle Vennemann, Christa Augustin

**Affiliations:** 1 Institute of Legal Medicine, University Medical Center Hamburg–Eppendorf, Martinistraße 52, 20246 Hamburg, Germany; 2 Institute of Legal Medicine, University of Münster, Röntgenstraße 23, 48149 Münster, Germany; 3 Institute of Biostatistics and Clinical Research, University of Münster, Münster, Germany

**Keywords:** Age prediction, DNA methylation, Forensic phenotyping, Minisequencing, Buccal swab

## Abstract

DNA methylation patterns change during human lifetime; thus, they can be used to estimate an individual’s age. It is known, however, that correlation between DNA methylation and aging might not be linear and that the sex might influence the methylation status. In this study, we conducted a comparative evaluation of linear and several non-linear regressions, as well as sex-specific versus unisex models. Buccal swab samples from 230 donors aged 1 to 88 years were analyzed using a minisequencing multiplex array. Samples were divided into a training set (*n* = 161) and a validation set (*n* = 69). The training set was used for a sequential replacement regression and a simultaneous 10-fold cross-validation. The resulting model was improved by including a cut-off of 20 years, dividing the younger individuals with non-linear from the older individuals with linear dependence between age and methylation status. Sex-specific models were developed and improved prediction accuracy in females but not in males, which might be explained by a small sample set. We finally established a non-linear, unisex model combining the markers EDARADD, KLF14, ELOVL2, FHL2, C1orf132, and TRIM59. While age- and sex-adjustments did not generally improve the performance of our model, we discuss how other models and large cohorts might benefit from such adjustments. Our model showed a cross-validated MAD and RMSE of 4.680 and 6.436 years in the training set and of 4.695 and 6.602 years in the validation set, respectively. We briefly explain how to apply the model for age prediction.

**Supplementary Information:**

The online version contains supplementary material available at 10.1007/s00414-023-02967-6.

## Introduction

If the source of a biological trace cannot be identified by conventional DNA comparison, forensic DNA phenotyping (FDP) of biological traces might provide further investigative leads. Information on phenotypical aspects of the donor of a trace, such as skin, eye and hair color, height, or even male baldness patterns [1–3], might help narrowing down the group of potential trace donors. While such characteristics are mainly determined by single nucleotide polymorphisms (SNPs), a trace donor’s age can be estimated based on epigenetic modifications such as age correlated DNA methylation [4, 5].

Beside analyzing DNA traces, further potential fields of applying molecular age estimation comprise the identification of unknown bodies [6, 7] and the objective confirmation of age in potentially underaged individuals: in many countries, unaccompanied underaged refugees are entitled to special protection, and objective age estimation can support such claims.

Recent studies revealed high estimation accuracy of age estimation models for blood with a MAD ranging from 3.16 to 10.33 years [8–13]. Best correlations and the lowest estimation errors were found for blood, buccal epithelium, and saliva among other tissues [14]. Currently, most forensic studies on age correlated methylation patterns and model validation are based on blood, while for saliva and buccal swabs, fewer models have been described. Estimation accuracies for saliva and buccal swabs are comparable to those for blood with MAD ranging from 3.13 to 5.8 years and 3.22 to 5.33 years, respectively [13, 15–19].

Several recent studies focused on methylation patterns of young individuals [20, 21]. While there is a significant overlap between age-associated methylation loci between adults and children, DNA methylation in children changes with up to fourfold higher rates compared to adults [20]. Furthermore, correlation between methylation and age is not always linear but might be logarithmic [20]. Several studies made similar conclusions describing that DNA methylation alters at a more rapid pace between childbirth and adolescence compared to adulthood [22, 23].

The aim of this study was to develop an age estimation model and analyze whether or not considering varying methylation change rates in young versus older individuals improves the prediction model. Human oral mucosa samples were analyzed by minisequencing multiplex PCR. The eight markers used in the study (PDE4C, EDARADD, SST, KLF14, ELOVL2, FHL2, C1orf132, and TRIM59) have been previously reported separately to show a correlation with age [6, 8, 15, 18, 19, 24, 25]. Especially ELOVL2, KLF14, and TRIM59 have been described as highly accurate markers for age estimation [15, 26].

## Material and methods

### Sampling, DNA extraction, and quantification

Oral mucosa samples from 230 donors (102 male and 128 female) aged 1 to 88 years (mean 38 years) were collected using sterile swabs. The Ethics Committee of the Hamburg Medical Association (Ethikkommission bei der Bundesärztekammer) approved the study protocol (PV6098) and all participants or their legal representatives provided written informed consent. DNA was extracted using the Casework Extraction Kit and Maxwell 16 (Promega) following manufacturer’s recommendations. DNA was quantified using the PowerQuant System (Promega) following manufacturer’s recommendations. Purified DNA samples were stored at 6 °C until further use.

### Bisulfite conversion

DNA samples were bisulfite converted and purified following the instructions of the EpiTect Fast DNA Bisulfite Kit (Qiagen) for high concentration samples. Depending on the determined concentration of each sample, up to 400 ng DNA was used for the treatment. Carrier RNA was not added to Buffer BL. Unmethylated cytosines were converted to uracils by bisulfite treatment, whereas methylated cytosines remained unconverted. To prove a successful bisulfite conversion, a second PowerQuant reaction was performed. The PCR primers should not bind to the converted DNA, meaning that a negative result would prove a complete conversion [19]. Bisulfite converted samples were stored at 6 °C.

### PCR and minisequencing multiplex

The bisulfite converted DNA was amplified by PCR using the PyroMark PCR Kit (Qiagen). Each sample was set in three reactions with primers for eight different markers (for primer sequences see Supplementary Table 1); first reaction contained primers for PDE4C, EDARADD, SST, and KLF14, second reaction contained primers for ELOVL2 and C1orf132, and third reaction contained primers for FHL2 and TRIM59. After PCR, 1.25 μl rAPid Alkaline Phosphatase (1 U/μl, Roche) and 0.025 μl Exonuclease I (20 U/μl, Thermo Fisher Scientific) were added to each sample for enzymatic digestion. The samples were incubated for 1 h 35 min at 37 °C followed by denaturation for 15 min at 78 °C. For differentiation between cytosines (methylated) and thymines (originally unmethylated cytosines), a minisequencing reaction was conducted using SNaPshot Multiplex Kit (Thermo Fisher Scientific). Following the sequencing reaction, another 1 μl rAPid Alkaline Phosphatase (1 U/μl, Roche) was added to each sample for enzymatic digestion. The samples were incubated for 1 h 15 min at 37 °C and 15 min at 78 °C and afterwards stored at 6 °C.

### Capillary electrophoresis and analysis

Samples were analyzed by capillary electrophoresis on a 3130 Genetic Analyzer (Applied Biosystems). Size standard 120 LIZ (Applied Biosystems); diluted 1:100 in HiDi formamide (Applied Biosystems) was used and results were evaluated using Gene Mapper ID (v3.2). The proportion of methylated cytosines of the samples was determined by calculating the relative peak heights for adenine and guanine or thymine and cytosine, respectively.

### Statistics

Correlations between chronological age and methylation status of each CpG site was assessed calculating Pearson correlation coefficient (*r*) and corresponding *p* values.

Data was split into a training and validation set, comprising 161 and 69 samples, respectively. Model accuracy was tested for both, training and validation set using the coefficient of determination *R*^2^, adjusted *R*^2^ value. Mean average deviation (MAD) and the root-mean-square error (RMSE) were computed on the training set (via cross-validation) as well as on the validation set. Statistical analyses were performed using R (version R-4.1.2) including the packages *ggplot2* [27] and *gridExtra* [28] for the creation of figures, Microsoft Office Excel 2016, and IBM SPSS Statistics 25 (IBM Corporation, Somers, NY, USA).

To make it easier to follow, a detailed description of model development and validation regarding non-linear dependences and influence of the sex can be found in the “Results and discussion” section.

## Results and discussion

**Fig. 1:**
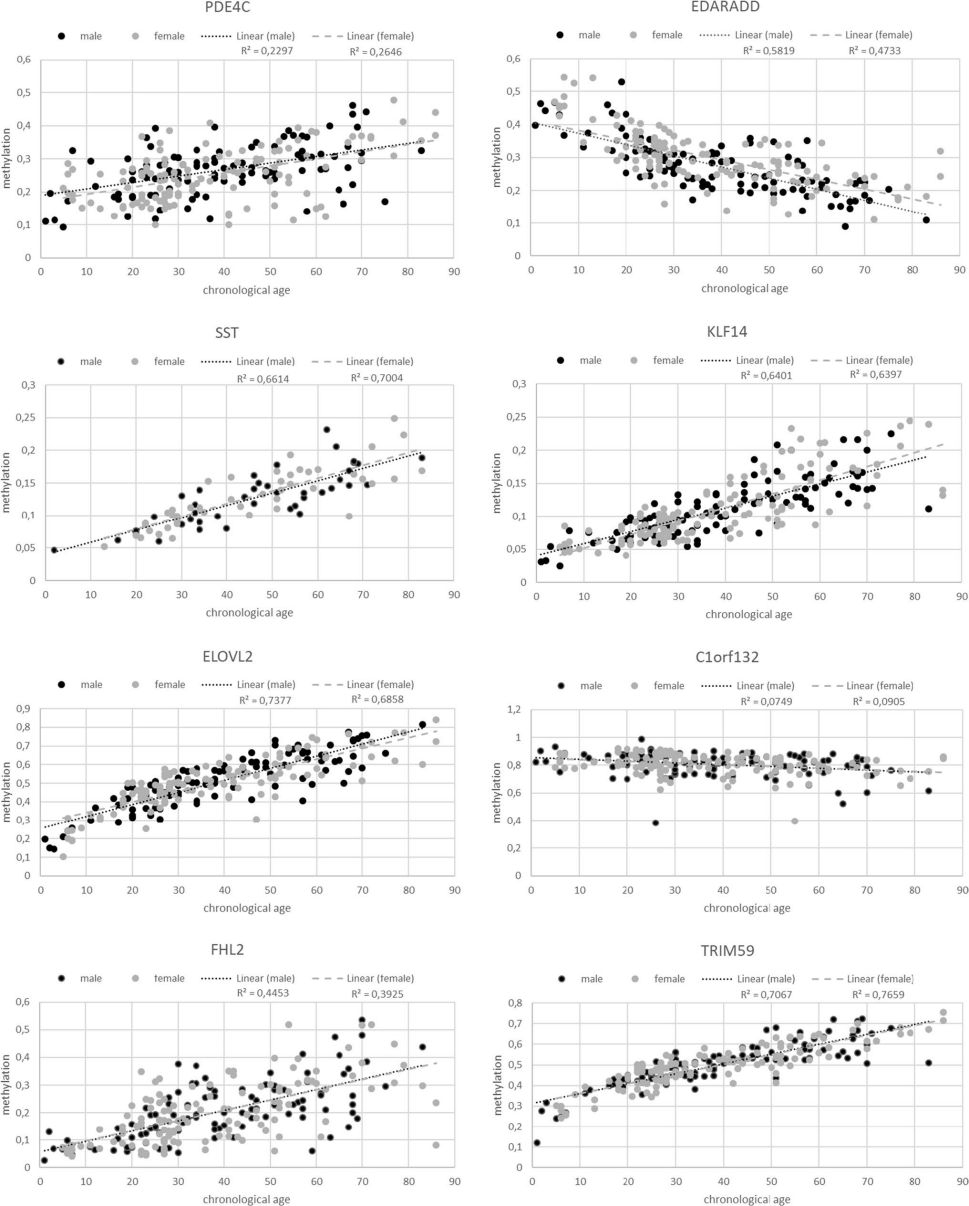
Correlation between chronological age and methylation at the eight CpG sites in all 230 buccal swap samples

Examples of the multiplex results are shown in Supplementary Figure 1. The correlation between chronological age and methylation status of each CpG site was assessed using *R*^2^ (Fig. 1), Pearson correlation coefficient (*r*), and corresponding *p* values (Supplementary Table 2). There were statistically noticeable correlations between chronological age and the methylation status at seven of the eight CpG sites (PDE4C, EDARADD, SST, KLF14, ELOVL2, FHL2, and TRIM59). The strongest correlation was detected for the CpG site in TRIM59 (*r* = 0.86). In this study, SST, ELOVL2, and TRIM59 revealed the strongest correlations with age, matching the results of previous studies [15, 18, 26]. In contrast, PDE4C (cg17861230 +36 bp) showed weaker correlations with age compared to a previous study [19]. EDARADD and C1orf132 were the only markers in this study showing negative correlations with age. In previous studies [15, 19], SST cg00481951 was a promising marker for age estimation and was incorporated into the model of Hong et al. [15]. Although SST showed moderate to good correlation and *R*^2^ values in our study, it had to be removed from further analysis due to missing values from two thirds of all samples.

### Model construction and validation

Due to missing values in the data set of SST, this marker was excluded. The rest of the markers showed moderate to strong correlations with chronological age; therefore, the seven CpG sites of the markers PDE4C, EDARADD, KLF14, ELOVL2, FHL2, C1orf132, and TRIM59 of 161 individuals (training set) were included in the regression analysis.

It is well known that the relationship between methylation and chronological age is not necessarily linear [14, 29]. Our methylation data shown in Fig. 1 also raises this suspicion. In particular, the epigenetic age advances faster during adolescence (age ≤ 20) and slower for elderly people (age ≥ 80) compared to chronological age. In between, epigenetic and chronological age are assumed to have a linear relationship (see Figure 1c in [29]). As the amount of elderly people with an age over 80 is rather small in our data set (3 subjects in the training data set and 1 subject in the validation data set), we focus on the non-linear relationship for adolescents. The following transformation has been suggested to model the described behavior by connecting chronological age *y*_*c*_ and epigenetic age *y*_*e*_ and improve the performance of subsequent regression analyses [14]:

$$f\left({y}_c;\;{y}_{c,adult}\right):= \left\{\begin{array}{c}\log \left({y}_c+1\right)-\log \left({y}_{c, adult}+1\right)\\ {}\\ {}\frac{y_c-{y}_{c, adult}}{y_{c, adult}+1}\end{array}\kern2em \genfrac{}{}{0pt}{}{\textrm{if}\ {y}_c\le {y}_{c, adult}}{\begin{array}{c}\\ {}\textrm{else}\end{array}}\right.$$

**Fig. 2:**
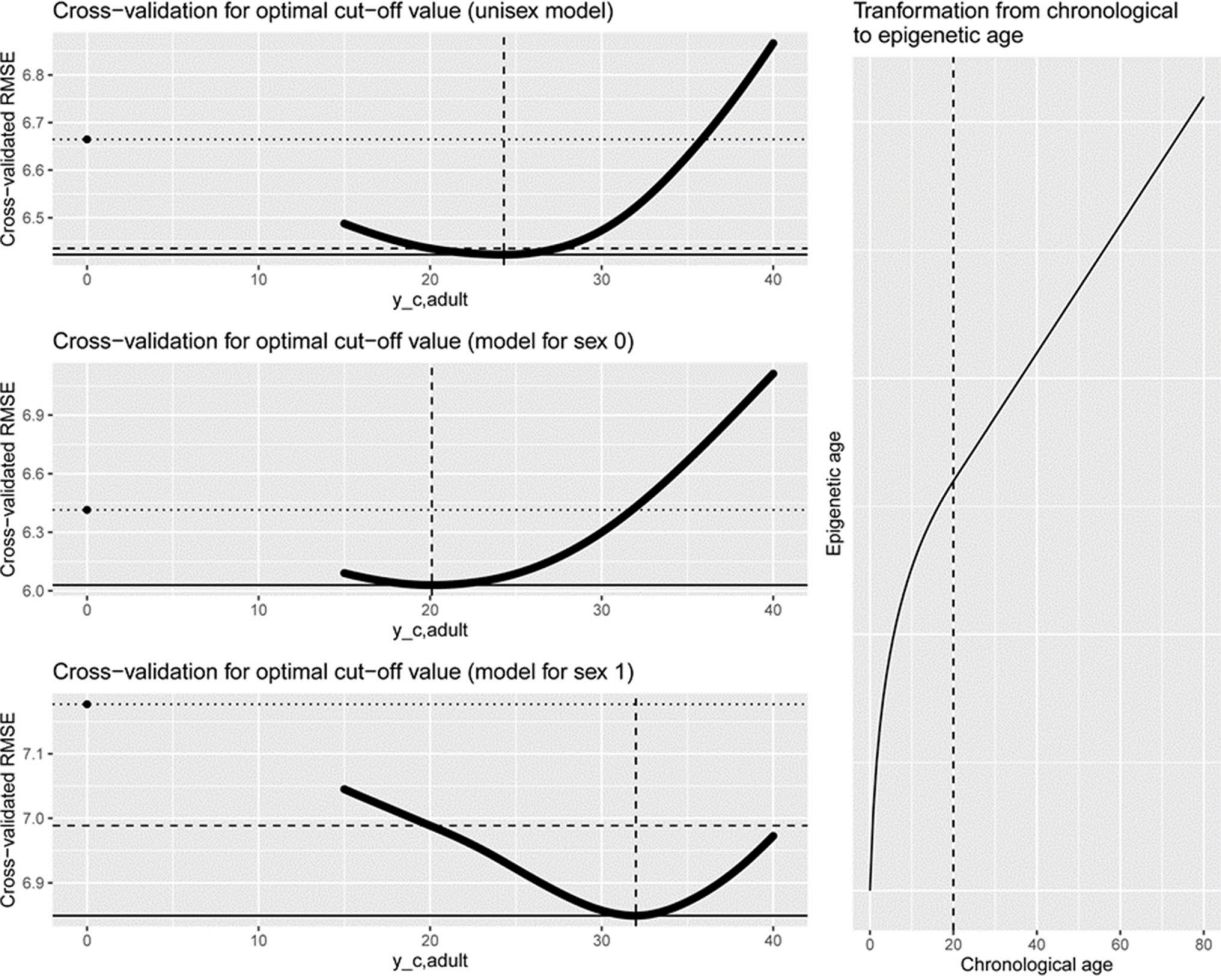
On the left: RMSE from repeated cross-validation of age prediction models on the training set given different cut-off values for a unisex model (first row), a model for sex 0 = men (second row), and a model for sex 1 =women (third row); vertical line indicates the cut-off value achieving the smallest RMSE; horizontal lines indicate RMSEs for the standard linear model (dotted), the default cut-off value (dashed), and the minimal value (solid). On the right: shape of the transformation function linking chronological and epigenetic age with the standard cut-off (vertical line)

This transformation is also displayed for *y*_*c*, *adult*_ = 20 on the right hand side of Fig. 2. Larger values of *y*_*c*, *adult*_ lead to even steeper curves at the origin. For a fixed value of *y*_*c*, *adult*_, one may then conduct a regression analysis to estimate regression parameters *β* for the linear model:

$$f\left({y}_c;{y}_{c,adult}\right)=: {y}_e={\beta}_0+{\beta}_1{x}_1+\dots +{\beta}_k{x}_k+\varepsilon$$

where *x*_1_, …, *x*_*k*_ denote methylation values from *k* different CpG sites. The estimated chronological age can be identified afterwards by application of the inverse of *f* for a fixed value of *y*_*c*, *adult*_, where $\hat{\beta}$ denotes the estimated regression parameters:

$${\hat{y}}_c:= {f}^{-1}\left({\hat{y}}_e;{y}_{c,adult}\right)={f}^{-1}\left({\hat{\beta}}_0+{\hat{\beta}}_1{x}_1+\dots +{\hat{\beta}}_k{x}_k;{y}_{c,adult}\right)$$

Upon first-time application of this transformation [14], the value *y*_*c*, *adult*_ was set to 20. Since a study on further choices of this value has not been described in the original work by Horvath, we investigated whether the choice of this cut-off value can be optimized. That is, we regard the cut-off *y*_*c*, *adult*_ from Horvath’s transformation as a hyperparameter whose value shall be determined via a repeated 10-fold cross-validation on the training data. We repeated this cross-validation 100 times to exclude a dependence of the results from the choice of the folds.

Furthermore, we investigated potential sex-specific differences in epigenetic development. As sex-specific influences on age estimation models were discussed in previous studies [11, 19, 30], we also examined these effects in our data. Therefore, we repeated the procedure described above for the subsets of data consisting only of women resp. men to find sex-specific differences. That resulted in different cut-off values *y*_*c*, *adult*_ for women and men. The results of the cross-validation procedure on the training data set can be found in Fig. 2. While the cross-validated RMSE indicates that the choice of *y*_*c*, *adult*_ = 20 is a good choice for a unisex model and a separate model for men, the RMSE for a separate model for women can be improved by choosing a larger cut-off value. This suggests that there are differences in the epigenetic aging pattern between men and women [14] which give rise to establishing sex-specific models to improve prediction, especially for adolescents and young adults. The exact values for the minimal RMSE and the corresponding cut-off value of the cross-validation can be found in Table 1. Please note that a cut-off value of 0 corresponds to the standard linear model.

**Table 1:** Results of repeated cross-validation (columns 3–5) and validation on separate set (columns 6–8). First three rows show results of standard linear regression, rows 4–6 show results of model with transformation based on default cut-off of 20, and last three rows show results of models in which the cut-off point was chosen based on the cross-validation on training set. Models in rows 1, 4, and 7 were fitted based on data from females and males; other models were only fitted based on the respective sex-specific subsets

Model	*y*_*c,adult*_	RMSE in cross-validation			RMSE on validation set		
		All samples (*n* = 161)	Men (*n* = 73)	Women (*n* = 88)	All samples (*n* = 69)	Men (*n* = 29)	Women (*n* = 40)
Linear	0	6.6646	5.8977	6.9222	6.8551	7.0728	6.6928
Linear, men	0	–	6.4145	–	–	7.4086	–
Linear, women	0	–	–	7.1769	–	–	6.6804
Default	20	6.4356	5.6179	6.7257	6.6018	7.3008	6.0446
Default, men	20	–	6.0293	–	–	7.8691	–
Default, women	20	–	–	6.9886	–	–	6.1999
CV, unisex	24.3	6.4225	5.6144	6.7177	6.6831	7.4848	6.0356
CV, men	20.1	–	6.0293	–	–	7.8757	–
CV, women	32.0	–	–	6.8490	–	–	6.1775

Finally, we fitted the different models on the training data resp. its sex-specific subsets and computed the RMSE on the validation set. While the observations from the cross-validation could be replicated for women, this was not possible for the unisex model (where the optimized model performed worse than the default choice) and men (where both transformations performed worse than the standard linear model). However, this may be due to the small sample sizes, especially for men with only 29 individuals in the validation set.

As shown in Fig. 2 and Table 1, our results suggest a faster epigenetic aging in men compared to women. These findings are concordant with the results of Hannum et al. [31]. Table 1 shows higher RMSE values for men on the validation set, which supports findings of previous studies [9, 11, 19].

As we are dealing with quite small sample sizes, the sex-specific models have a clear disadvantage in that they can only be fitted with half of the observations. This disadvantage should diminish with an increasing overall sample size [32]. In this light, based on the results obtained here, it appears reasonable to consider sex-specific models and respective transformations for estimating chronological age in future studies with larger sample sizes.

Age estimation of the training set revealed a strong correlation with age (*r* = 0.942) and a MAD and RMSE of 4.680 and 6.436 years, respectively. Within the training set, the seven CpG site model could explain 88.8% of the age variance (*R*^2^ = 0.888, adj. *R*^2^ = 0.883).

### Application of the model

In the present situation, it appears most appropriate to apply the unisex model with the default cut-off value of *y*_*c*, *adult*_ = 20 when the chronological age of new subjects shall be estimated. We give a brief outline of how the age estimation for an individual of unknown age can be performed by applying this model with methylation values *x*_*PDE*4*C*_, *x*_*EDARADD*, _ *x*_*KLF*4_, *x*_*ELOVL*2_, *x*_*FHL*2_, *x*_*C*1*orf*132_, and *x*_*TRIM*59_ (each between 0 and 1). Firstly, the linear prediction according to our estimation of the epigenetic age ${\hat{y}}_e$can be computed via:

$${\hat{y}}_e=-1.5880-0.0400\ {x}_{PDE4C}-1.9120\ {x}_{EDARADD}+5.0157\ {x}_{KLF4}+0.5961\ {x}_{ELOVL2}+1.7463\ {x}_{FHL2}-0.0108\ {x}_{C1 orf132}+3.5634\ {x}_{TRIM59}$$

If this value is positive, the chronological age can be transformed back to the chronological scale with the linear transformation:

$${\hat{y}}_c={f}^{-1}\left({\hat{y}}_e;\;20\right)={\hat{y}}_e\ast \left(20+1\right)+20$$

Otherwise (if values are negative), one obtains the estimated chronological age via:

$${\hat{y}}_c={f}^{-1}\left({\hat{y}}_e;\;20\right)=\exp \left({\hat{y}}_e+\log \left(20+1\right)\right)-1$$

Prediction intervals for the chronological age can be computed from the same backtransformation procedure after computing prediction intervals on the epigenetic scale with the model’s residual standard error of 0.2905. One should note that the shape of the backtransformation function leads to shorter prediction intervals for subjects that are estimated to be young.

### Model performance on the validation set

**Fig. 3:**
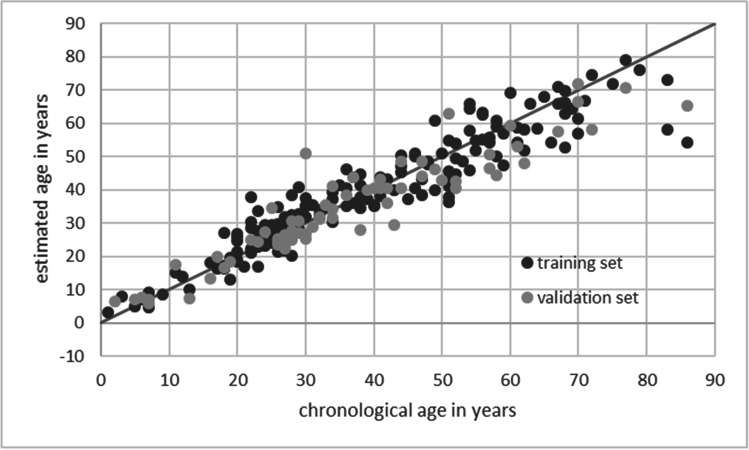
The seven CpG site age estimation model predicting age for both training and validation set

**Fig. 4:**
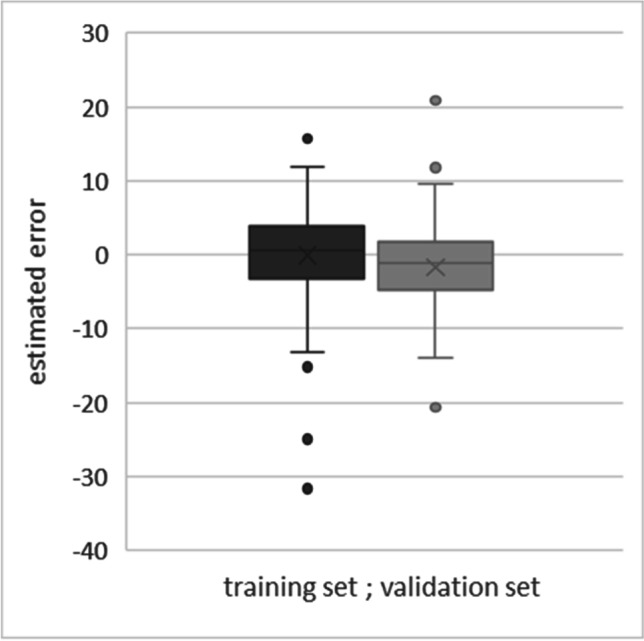
Deviation of estimation errors from chronological age for the training and validation set. Boxplots represent the estimation error deviation for the training and validation set. Circles represent outliers in the corresponding set

**Fig. 5:**
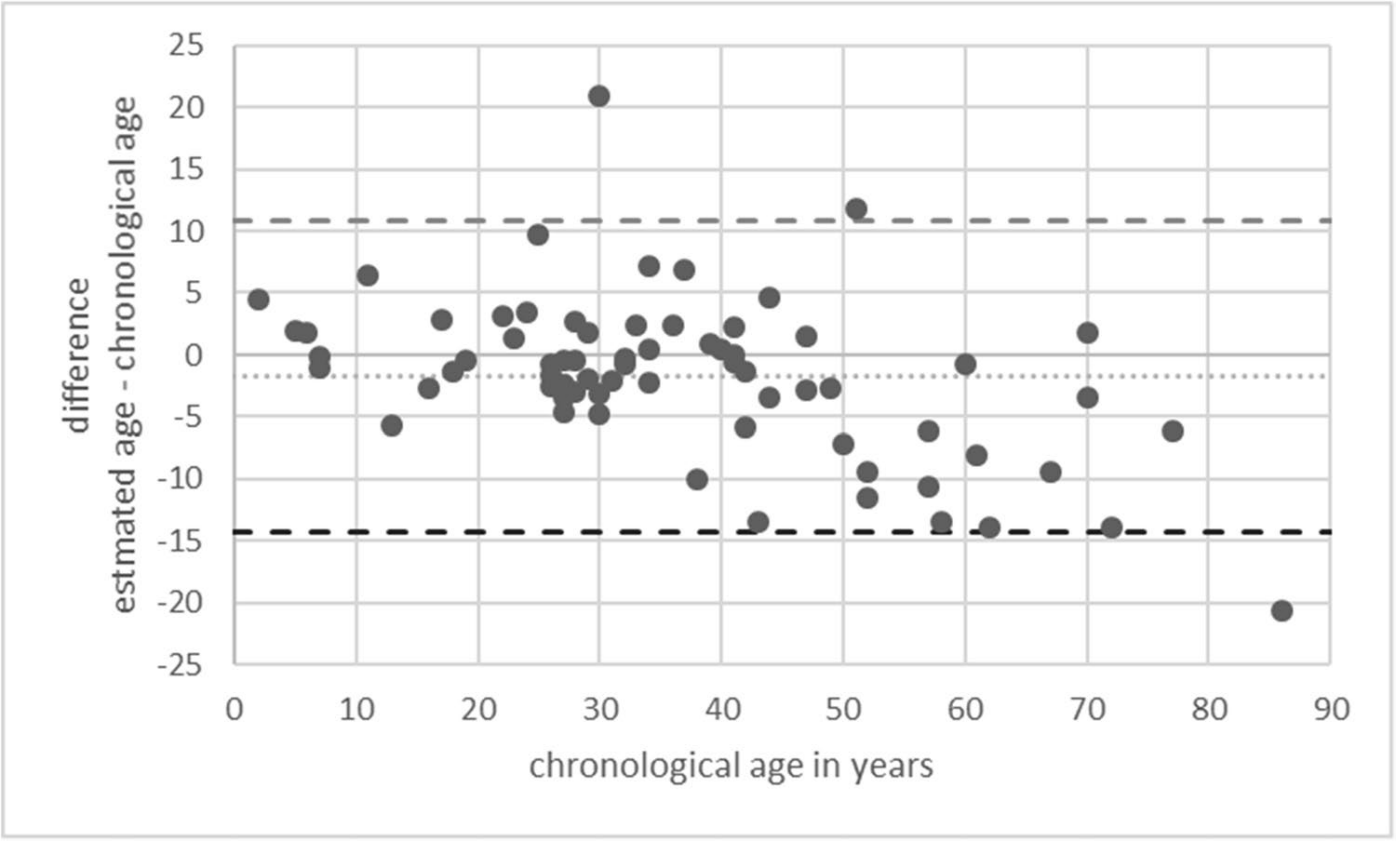
Agreement between estimated and chronological age in the validation set. The chronological age was plotted against the difference between estimated age and chronological age. Each data point represents one analyzed sample within the validation set (*n* = 69). The dotted line represents the mean difference; the dashed lines show the upper and lower limits of agreement (1.96 × SD and −1.96 × SD, respectively). SD standard deviation

The resulting model comprised seven CpG sites of the markers PDE4C, EDARADD, KLF14, ELOVL2, FHL2, C1orf132, and TRIM59, explaining 87.8% of age variance in the validation set (*R*^2^ = 0.878, adj. *R*^2^ = 0.864). The age predictions of the resulting model have a strong correlation between chronological and predicted age (*r* = 0.937) with a MAD of 4.695 years and a RMSE of 6.602 years (Fig. 3). As seen in Fig. 3, age predictions of training and validation sets showed a similarly strong correlation between predicted and chronological age. The high comparability of the training and validation set is also shown in Fig. 4, which compares the estimation errors of both data sets. Further visualization of the difference between chronological and estimated age is shown in the Bland-Altman plot in Fig. 5. A mean difference of −1.718 (SD 6.417) years indicates a slight underestimation of age. The 95% limit of agreement ranges from 10.86 to −14.295 years. The plot shows a tendency to overestimate younger individuals, especially from 0 to 20 years, and to underestimate older individuals (50+ years). Studies from Naue et al. [26] and Schwender et al. [19] made similar observations. The largest positive deviation from chronological to estimated age within the validation set (20.904 years) was found for a 30-year-old individual with an estimated age of 50.904 years. The largest negative deviation (−20.685 years) was found for an 86-year-old individual with an estimated age of 65. 315 years. To further assess model performance, we followed the recommendations of Schwender et al. [33]. The validation set was subdivided into age categories and the absolute deviation between estimated and chronological age was split into four categories (up to ±3 years deviation, up to ±4 years, up to ±5 years, and up to ±6 years deviation) as shown in Table 2. In general, prediction accuracy in younger individuals was higher compared to older individuals. Similar tendencies were observed regarding the MAD, confirming results of previous studies [9, 18, 26, 30, 34].

**Table 2:** Age estimation for different age ranges: the first row shows the subdivided groups by age. The second and third line the MAD of the subgroups and the number of individuals in the subgroup. The following rows showing the percentage of age estimations within given error range of ±3 years, ±4 years, ±5 years, and ±6 years

Age range (years)		0–19	20–39	40–59	60–90	All
MAD (years)		2.64	3.59	5.74	8.68	4.69
*n*		11	30	19	9	69
Percentage of age estimations within given error range	±3 yrs	72.7	60.0	42.1	22.2	52.2
	±4 yrs	72.7	76.7	47.4	22.2	62.3
	±5 yrs	81.8	83.3	52.6	22.2	68.1
	±6 yrs	81.8	83.3	57.9	22.2	71.0

The study presented here comprises two obvious shortcomings: the number of individuals ages 60+ was rather small within our validation set. Consequently, prediction accuracy of the model cannot be reliably assessed within this age group. Secondly, environmental influences have not been taken into account in this study, even though they might play a role in the changing of DNA methylation patterns [34].

## Conclusion

The main aim of this study was to evaluate a set of CpG sites as reliable DNA methylation predictors of chronological age in minors as well as in adult individuals and different sexes by performing a minisequencing multiplex assay. Seven CpG sites (cg17861230 (+36 bp), cg09809672 (−12 bp), cg14361627, cg16867657 (−16 bp), cg06639320, cg10501210 (+6 bp), and cg07553761) in EDARADD, KLF14, ELOVL2, in FHL2, in C1orf132, and TRIM59 were included in this study. Validation of the final model revealed a cross-validated MAD and RMSE of 4.680 and 6.436 years in the training set and 4.695 and 6.602 years in the validation set, respectively, making this model likely to be useful in forensic investigations in the future. Regarding RMSE, sex-specific models did not outperform the unisex models in our limited data set. In larger sample sets, however, sex-specific modeling might increase prediction accuracy. DNA methylation analysis by minisequencing has the potential to become a tool in criminal investigation. Compared to massively parallel sequencing approaches, minisequencing has the benefit of being more flexible, less time consuming when analyzing small sample numbers, and easy to implement into forensic laboratories without the need for specified sequencing equipment.

## Supplementary Information

ESM 1 (PDF 209 kb)

ESM 2 (XLSX 11 kb)

ESM 3 (XLSX 41 kb)
