# Supplementary figures and images for: DNA methylation-based age estimation for adults and minors: considering sex-specific differences and non-linear correlations
Source: Int J Legal Med. 2023 Feb 22;137(3):635–43. doi: 10.1007/s00414-023-02967-6 (PMC10085938; doi:10.1007/s00414-023-02967-6)

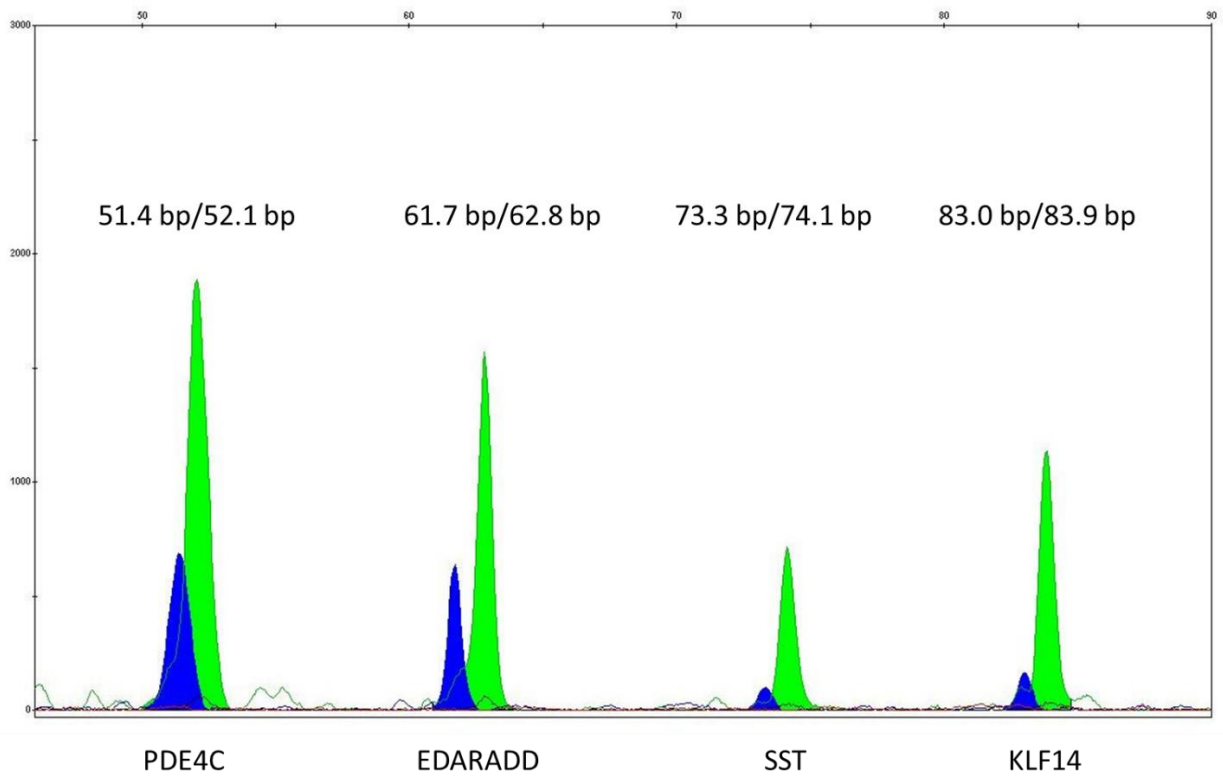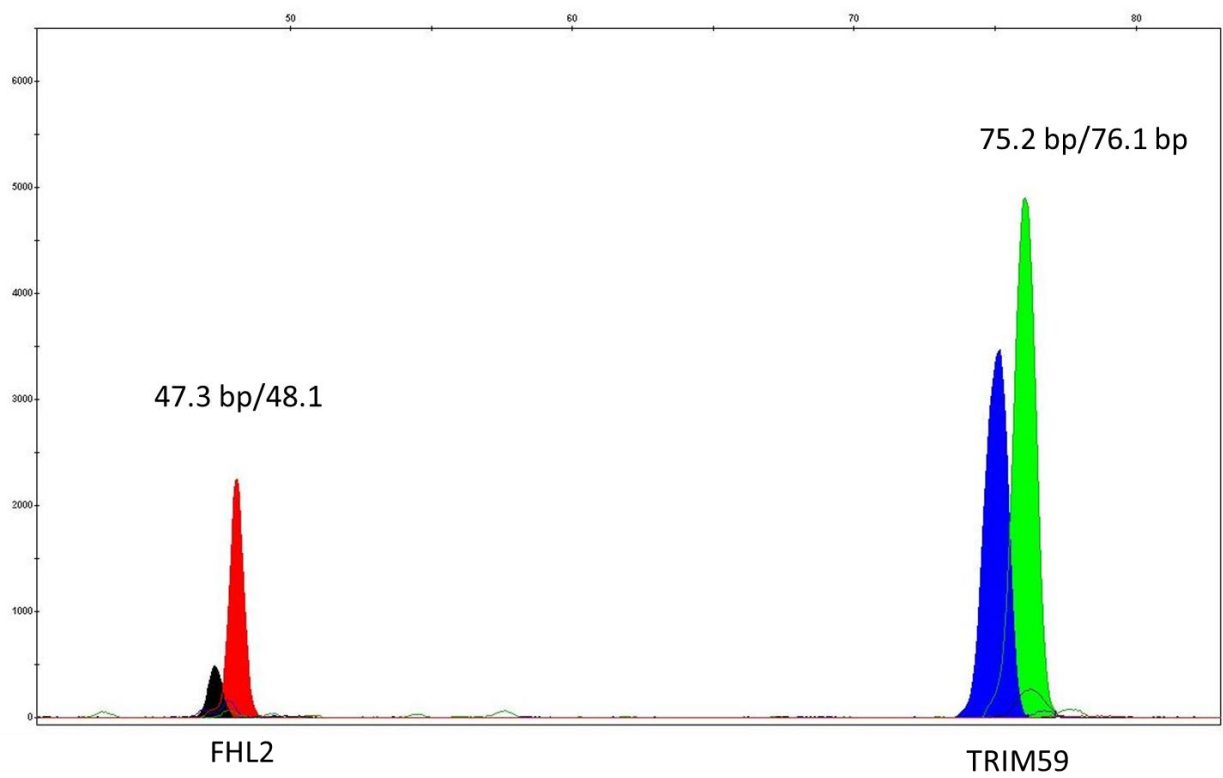

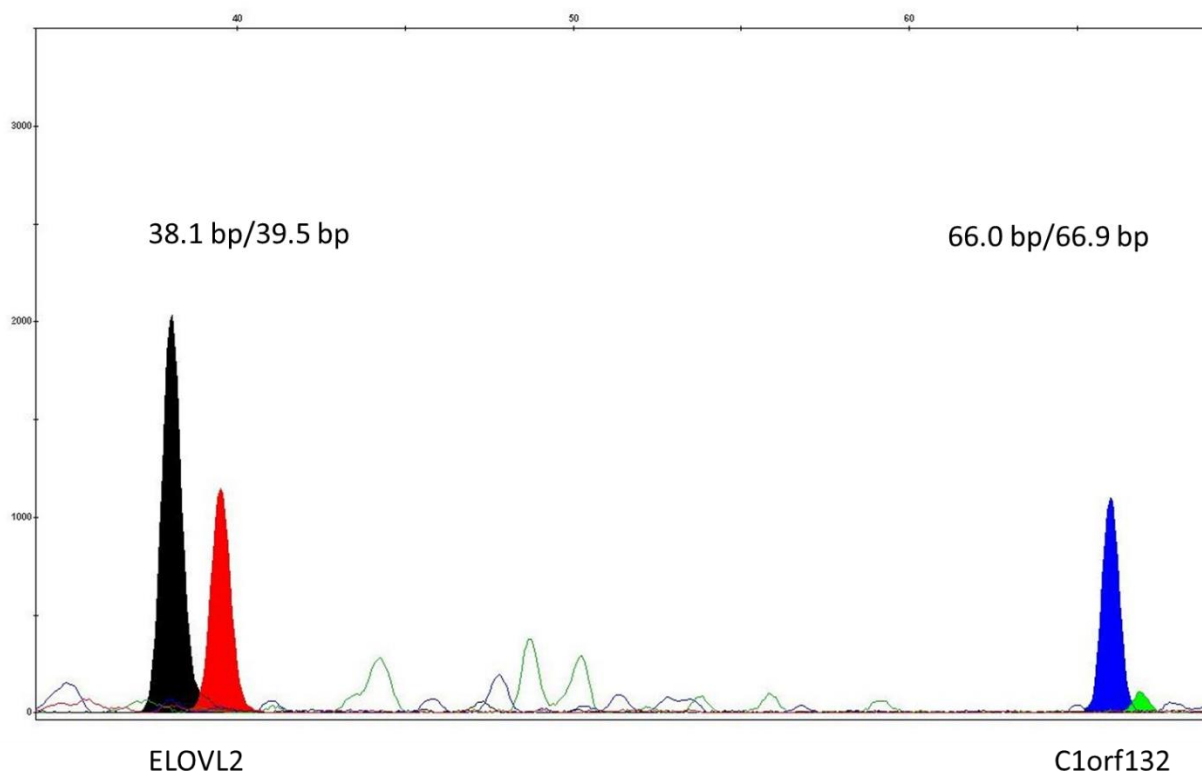

Supplement: Supplementary file 1 — (PDF 209 kb) [file 414_2023_2967_MOESM1_ESM.pdf]
